# Clinical validation of MR imaging time reduction for substitute/synthetic CT generation for prostate MRI-only treatment planning

**DOI:** 10.1007/s13246-023-01268-x

**Published:** 2023-05-23

**Authors:** Tony Young, Jason Dowling, Robba Rai, Gary Liney, Peter Greer, David Thwaites, Lois Holloway

**Affiliations:** 1 Liverpool and Macarthur Cancer Therapy Centres and Ingham Institute, Sydney, Australia; 2 Institute of Medical Physics, School of Physics, University of Sydney, Sydney, Australia; 3 CSIRO Health and Biosecurity, The Australian e-Health & Research Centre, Brisbane, QLD Australia; 4 South Western Sydney Clinical School, University of New South Wales, Sydney, NSW Australia; 5 School of Mathematical and Physical Sciences, University of Newcastle, Callaghan, NSW Australia; 6 Centre for Medical Radiation Physics, University of Wollongong, Wollongong, NSW Australia; 7 Calvary Mater Newcastle Hospital, Newcastle, NSW Australia

**Keywords:** MRI-Only, sCT, Prostate

## Abstract

Radiotherapy treatment planning based only on magnetic resonance imaging (MRI) has become clinically achievable. Though computed tomography (CT) is the gold standard for radiotherapy imaging, directly providing the electron density values needed for planning calculations, MRI has superior soft tissue visualisation to guide treatment planning decisions and optimisation. MRI-only planning removes the need for the CT scan, but requires generation of a substitute/synthetic/pseudo CT (sCT) for electron density information. Shortening the MRI imaging time would improve patient comfort and reduce the likelihood of motion artefacts. A volunteer study was previously carried out to investigate and optimise faster MRI sequences for a hybrid atlas-voxel conversion to sCT for prostate treatment planning. The aim of this follow-on study was to clinically validate the performance of the new optimised sequence for sCT generation in a treated MRI-only prostate patient cohort. 10 patients undergoing MRI-only treatment were scanned on a Siemens Skyra 3T MRI as part of the MRI-only sub-study of the NINJA clinical trial (ACTRN12618001806257). Two sequences were used, the standard 3D T2-weighted SPACE sequence used for sCT conversion which has been previously validated against CT, and a modified fast SPACE sequence, selected based on the volunteer study. Both were used to generate sCT scans. These were then compared to evaluate the fast sequence conversion for anatomical and dosimetric accuracy against the clinically approved treatment plans. The average Mean Absolute Error (MAE) for the body was 14.98 ± 2.35 HU, and for bone was 40.77 ± 5.51 HU. The external volume contour comparison produced a Dice Similarity Coefficient (DSC) of at least 0.976, and an average of 0.985 ± 0.004, and the bony anatomy contour comparison a DSC of at least 0.907, and an average of 0.950 ± 0.018. The fast SPACE sCT agreed with the gold standard sCT within an isocentre dose of -0.28% ± 0.16% and an average gamma pass rate of 99.66% ± 0.41% for a 1%/1 mm gamma tolerance. In this clinical validation study, the fast sequence, which reduced the required imaging time by approximately a factor of 4, produced an sCT with similar clinical dosimetric results compared to the standard sCT, demonstrating its potential for clinical use for treatment planning.

## Introduction

Radiotherapy treatment planning based only on magnetic resonance imaging (MRI), or MRI-only radiotherapy, has recently become clinically achievable [1–4]. Treatment planning requires electron density information for dose calculation, which is conventionally acquired from computed tomography (CT), but which MRI scans do not provide directly. However, substitute, synthetic, or pseudo CT (sCT) scans generated from specific MRI sequences are able to provide electron density information [5–7]. These sCT scans are validated against CT scans and their use removes the need to acquire a pre-treatment CT scan.

There are various approaches which have been utilised for sCT generation, such as bulk density correction methods, atlas methods, deep learning algorithms or a combination of these approaches. Bulk density correction requires only segmentation of the MRI, with appropriate density values applied to these segmentations for dose calculation [8]. Atlas or multi atlas methodologies have been successfully utilised in the male pelvis [9, 10], where training sets of registered CT-MRI image pairs are used to produce an average CT-MRI atlas. The advantages of this method include a robustness to artefacts and intensity differences between images, and realistic anatomical deformation due to the use of prior training information, with the main disadvantage of the method being that images that fall outside the bounds of the atlas training data may be unable to be matched appropriately [11]. Commercially available sCT generation software has used a combination of atlas and bulk density assignment methods for prostate sCT generation [12–14]. Deep learning and artificial intelligence (AI) methods have also been utilised for prostate sCT generation, with these techniques able to be used in combination with others for image segmentation and tissue classification [15–19]; however they require a large amount of data and resources for training, but are much faster than the atlas method for sCT generation, taking typically in the order of seconds compared to minutes.

Within an MRI simulation session for MRI-only radiotherapy treatment planning, various MRI sequences may be captured for specific visualisation, such as fiducial marker identification, or target and organ at risk delineation purposes in addition to any particular sequence or sequences required for specific sCT generation methods. The MR imaging portion of the simulation session can take a significant amount of time [1, 14], especially when compared to a CT simulation session in which only a single CT scan may be required for both anatomical information and treatment planning. Any reduction in MR imaging time for a sequence would reduce the overall simulation time, and reduce the potential for patient motion or organ variation, in particular prostate motion, bladder filling and rectal and bowel gas changes over the simulation session [20–22], as well as reduce patient discomfort and increase MRI scanner utilisation [23]. Time reduction in MRI sequences however could impact image quality, reducing signal to noise ratio, image contrast and resolution [24–28], the effect of which should be considered in the application of each particular sequence. Additionally, to improve online adaptation of treatment plans for patient treatment on MRI linear accelerators, any time reduction which causes no change in treatment plan dosimetric quality would benefit the patient, reducing their treatment time and increasing patient comfort and tolerance for treatment [29–31].

Previously, a volunteer study was conducted investigating the effects of MRI sequence time reduction on sCT generation for prostate MRI-only treatment planning and to determine a suitable sequence [32]. This follow-up study aims to clinically apply and validate the previously determined optimal fast MRI sequence with a prospective patient cohort undergoing MRI-only radiotherapy treatment planning. The sCT generated from the new fast MRI sequence with clinical patient data is evaluated by comparison both anatomically and dosimetrically to the current established and validated sCT generation method utilising the standard MRI sequence. This study will determine whether the new fast sequence can be utilised clinically in sCT generation for future MRI-only radiotherapy.

## Method

Ten prostate radiotherapy patients were included in the study. These patients were recruited to the NINJA (Novel Integration of New prostate radiation therapy schedules with adjuvant Androgen deprivation) clinical trial (ACTRN12618001806257) which had local ethics approval (HREC/18/LPOOL/420), investigating stereotactic radiotherapy to the prostate comparing monotherapy against a virtual high dose rate brachytherapy boost regimen. This clinical trial also contains an MRI-only planning sub-study, demonstrating the ability to fully transition centres from CT- to MRI-based prostate radiotherapy planning, which these patients were enrolled into. Patients in this trial were prescribed either 40 Gy in 5 fractions or a stereotactic boost of 20 Gy in 2 fractions followed by a standard 36 Gy in 12 fractions, with treatment plans consisting of two VMAT arcs. The patients involved in the current study were part of the MRI-only planning sub-study, and scanned on a Siemens (Erlangen, Germany) Skyra 3T MRI with a flat radiotherapy couch and body coil mounted on coil mounts as per trial protocol. Patient age ranged from 60 to 72, and Body Mass Index (BMI) ranged from 23.1 to 32, with a mean of 27.1.

The standard planning MRI sequence as used for the clinical trial was a 3D T2-weighted isotropic SPACE (Sampling Perfection with Application optimised Contrasts using different flip angle Evolution) sequence which covered the entire pelvis, with scan limits from L5/S1 to the pubic symphysis. This sequence was previously validated against CT by Dowling et al. [11], and has an average scan time of 5 min and 4 s. A time-reduced MRI sequence was achieved by varying a combination of repetition time (TR), turbo factor, partial Fourier acceleration and parallel imaging acceleration, following the findings from the volunteer study [21], reducing the average scan time to 1 min and 19 s. The sequence parameters are displayed in Table 1, with further detail available in Young et al. [32].

**Table 1:** MRI sequence parameters which differed between the standard and fast T2 SPACE and the average scan time for each sequence

MRI Sequence	Average TR (ms)	Turbo factor	Partial Fourier	iPAT Acceleration Factor	Average Scan Time (min:sec)
Standard T2 SPACE	1700	80	7/8	4	5:04
Fast T2 SPACE	1200	120	6/8	6	1:19

MRI sequences were converted to sCT using a hybrid atlas-voxel method as described in Dowling et al. [11], with the converted Fast SPACE (F-sCT) compared to the Standard SPACE (S-sCT) conversion. Mean Absolute Error (MAE) for HU for the entire body, along with tissue and bones only was calculated by comparing the F-sCT to the S-sCT with the auto-segmented body and bone masks from the S-sCT. An anatomical comparison of the body and bone volumes between the generated sCT was completed considering volume differences, mean Hausdorff distance and Dice Similarity Coefficient (DSC) comparison.

Treatment planning for these patients was completed on the S-sCT using the Pinnacle Treatment Planning System (v16.21; Philips Healthcare, Andover, MA) utilising the auto-planning module for beam optimisation. Patient treatment plans met all trial guidelines, with each treatment plan consisting of two full Volumetric Arc Therapy (VMAT) treatment beams. Each patient’s corresponding clinically approved treatment plan was copied to the F-sCT and recalculated for comparison of isocentre point dose, a 1%/1mm global gamma comparison and DVH analysis of the PTV, bladder and rectum.

## Results

**Fig. 1:**
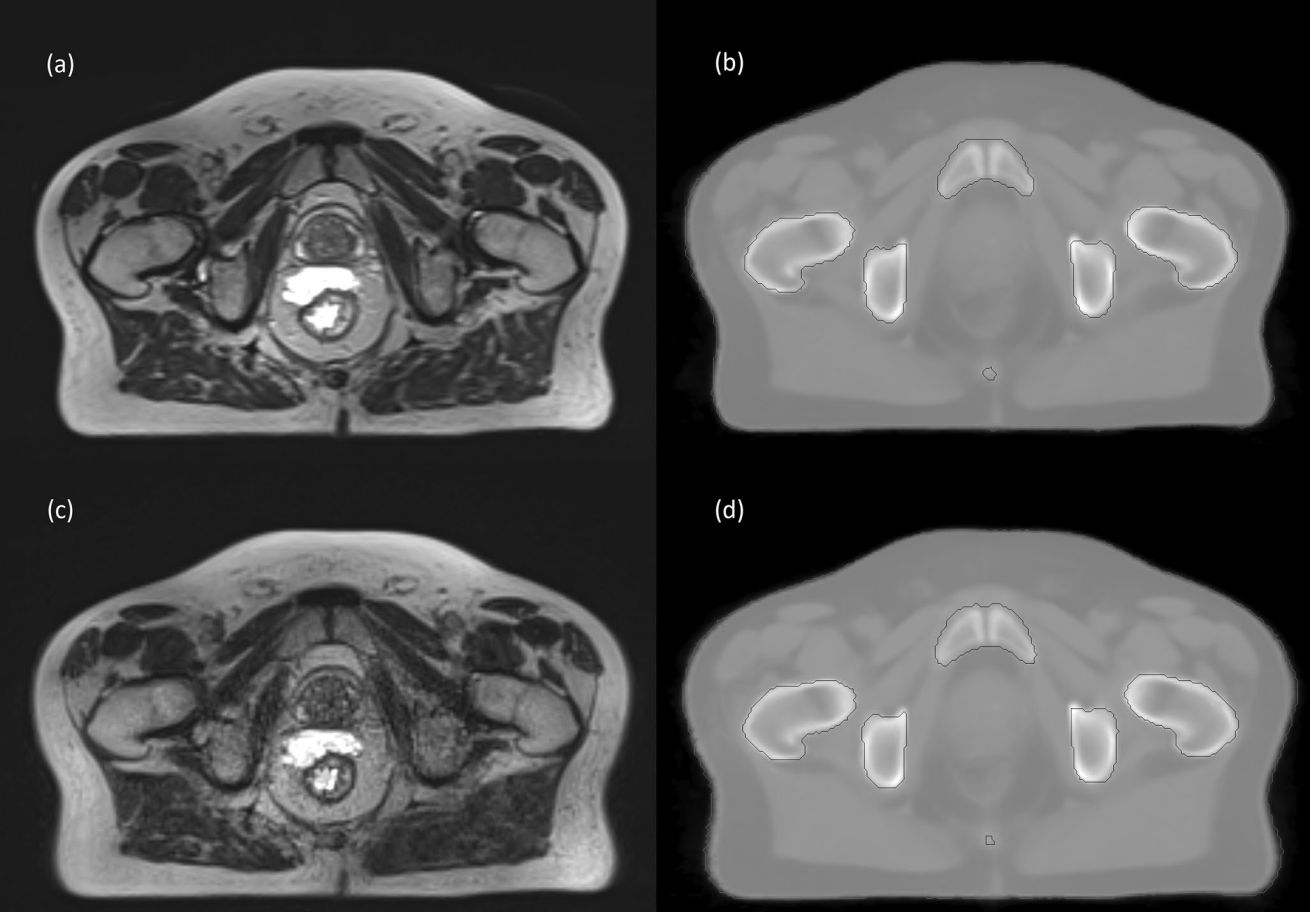
An example of the (a) standard MRI and (b) S-sCT and the (c) fast MRI and (d) F-sCT for the same corresponding slice for one patient. The body and bone masks are also displayed on the sCT slices in (b) and (d)

The Fast sequence scan was able to be completed on all patients with no modifications required by the system. This sequence was able to be converted to sCT as per Dowling et al. [11], with no additional artefacts seen in qualitative review of the fast MRI sequence scan or converted F-sCT. An example of the standard MRI and fast MRI, as well as the corresponding S-sCT and F-sCT can be seen in Fig. 1.

**Fig. 2:**
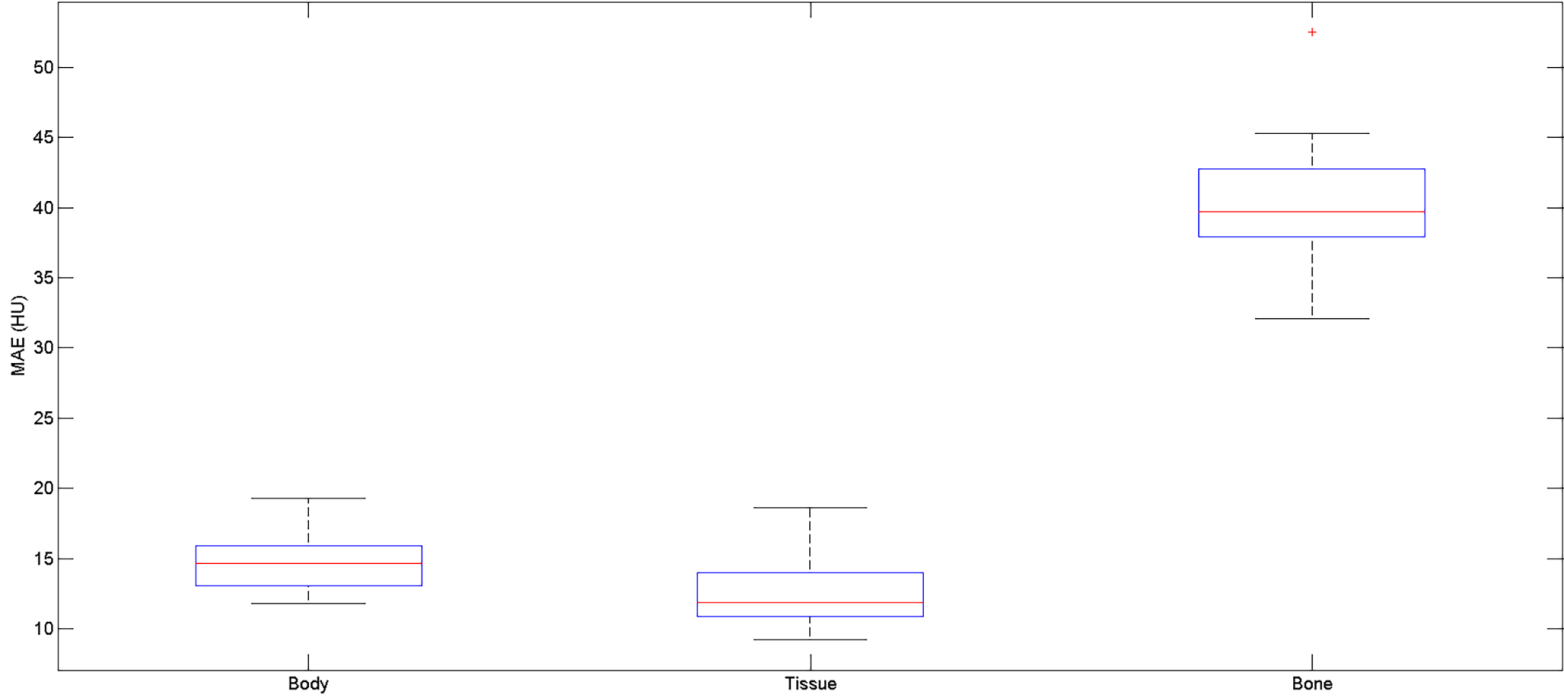
Mean Absolute Error (MAE) of HU for the generated sCT from the fast MRI sequence compared to the sCT generated from the standard MRI sequence for all patients for the within-the-body contour, the automatic bone contour, and for tissue only

The mean absolute error (MAE) in HU over the ten patients for the F-sCT compared to the S-sCT with the body and bone masks from the S-sCT, in addition to tissue only, is shown in Fig. 2. The average MAE for the body was 14.98 ± 2.35 HU, for tissue only was 12.68 ± 2.75 HU, and for the bone was 40.77 ± 5.51 HU.

**Table 2:** Volume percentage difference, mean Hausdorff distance and DSC results for automatic body and bone contours for the generated sCT for the fast MRI sequence compared to the standard sequence generated sCT for all patients

Patient	Body Volume % Difference	Body Mean Hausdorff (mm)	Body DSC	Bone Volume % Difference	Bone Mean Hausdorff (mm)	Bone DSC
1	-0.03%	0.76	0.990	-3.82%	0.71	0.948
2	3.99%	1.48	0.976	0.70%	1.29	0.907
3	2.26%	0.97	0.988	1.61%	0.43	0.967
4	1.13%	0.98	0.988	-0.25%	0.42	0.964
5	2.43%	1.24	0.986	-0.82%	0.71	0.937
6	0.22%	0.90	0.988	-5.23%	0.69	0.942
7	2.89%	1.24	0.984	0.98%	0.43	0.963
8	-1.17%	1.21	0.987	-2.65%	0.54	0.960
9	3.28%	1.47	0.982	1.79%	0.49	0.960
10	0.70%	1.65	0.981	0.74%	0.61	0.949

The volume percentage difference, mean Hausdorff distance and DSC results for both the body and bone contour comparison can be seen in Table 2. The average body volume difference was 1.57% ± 1.65%, whilst the average bone volume difference was − 0.69% ± 2.42%. The mean Hausdorff value was less than 2 mm for both the body and bone volumes, with the body contour comparison producing a DSC of at least 0.976, and an average of 0.985 ± 0.004, and the bone contour producing a DSC of at least 0.907, and an average of 0.950 ± 0.018.

**Table 3:** Dosimetric Results for all patient plan comparisons. The isocentre point dose was compared, as well as 1%/1 mm Global Gamma analysis for the clinical plan from the S-sCT recalculated on the F-sCT

Patient	Isocentre Point Dose	1%1 mm Gamma
1	-0.20%	99.10
2	-0.23%	98.87
3	-0.35%	99.56
4	-0.17%	99.89
5	-0.43%	100.00
6	-0.45%	99.39
7	-0.27%	99.93
8	0.07%	100.00
9	-0.42%	100.00
10	-0.31%	99.89

**Fig. 3:**
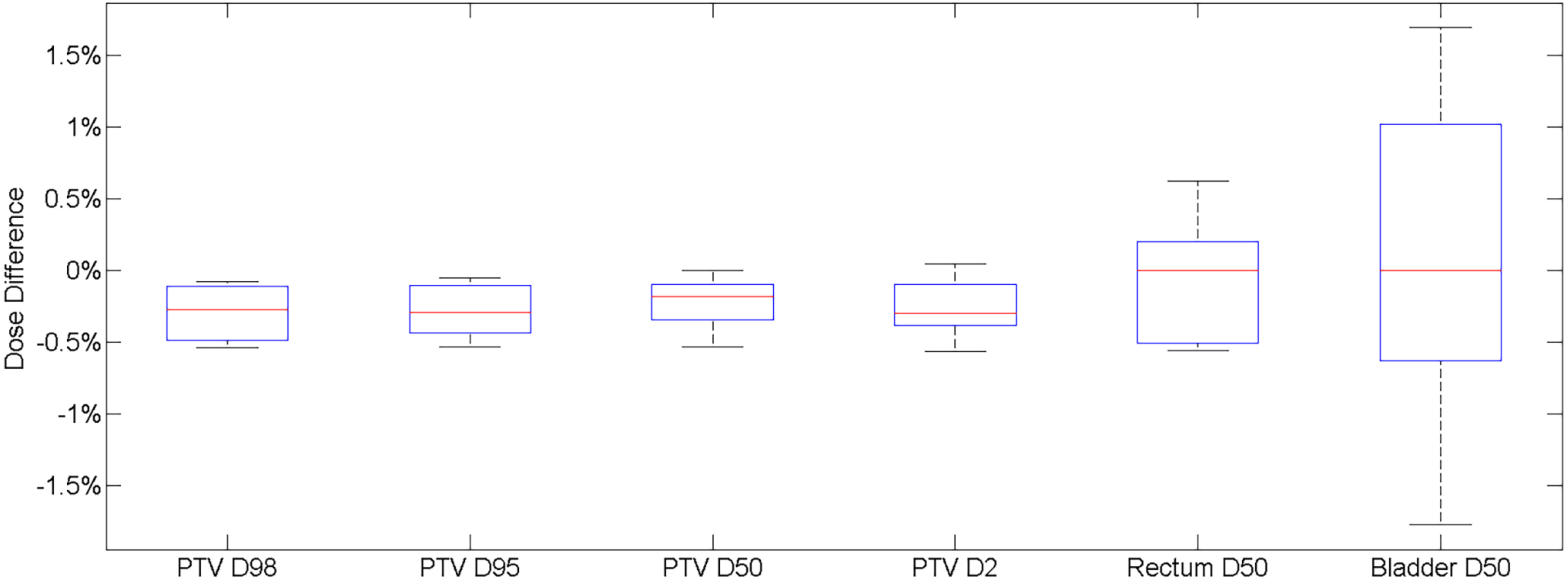
DVH comparison of PTV parameters and the rectum and bladder D50 for the recalculated treatment plans on the F-sCT compared to calculated on the S-sCT. This figure shows the percentage difference for each parameter for all patients

Table 3 shows dosimetric results. The isocentre point dose agreement for the clinical plan recalculated on the F-sCT was on average − 0.28% ± 0.16%, and within ± 0.5% of the S-sCT. The 1%/1 mm global Gamma pass rate was on average 99.66% ± 0.41%, with only one patient achieving below a 99% pass rate. The DVH dose differences are shown in Fig. 3. The PTV DVH statistics reported, on average, were within 0.5%, with an average difference of -0.27% ± 0.18%. The bladder and rectum D50 were within ± 2% on average, with the rectum D50 average difference being − 0.08 ± 0.40%, and the bladder D50 average difference being 0.08% ± 1.10%.

## Discussion

This study demonstrates clinical application and validation of the results from the previous volunteer study. It provides clinical data, using a patient cohort, regarding a time-reduced MRI sequence for sCT generation for prostate MR-only treatment planning. In an MRI-only workflow, additional sequences may be required for volume definition as image contrast and resolution may be enhanced or targeted to the anatomy, or functional sequences may provide additional guidance. The time reduction and associated reduction in image quality for the sCT sequence may be appropriate if anatomical and dose differences in the generated sCT for treatment planning are considered acceptable.

The potential trade-offs between reducing MR imaging time and the effects on the MR image quality and subsequent sCT generation accuracy should be considered [32]. A reduction in TR will produce an image with increased contrast between water and fat, being more T1-weighted [25]. An increase in the echo train length, the turbo factor, may increase the potential for artefacts, reducing signal-to-noise and image contrast and causing blurring in the image [33]. Changes in the partial Fourier factor may produce a time reduction in the scan by reducing the amount of k-space data acquired in the phase encoding direction, producing an image with a reduced signal-to-noise ratio [28]. Increasing the imaging acceleration factor will also alter the k-space data acquired, which may produce aliasing artefacts and reduce signal-to-noise [26, 34, 35]. Qualitative comparison of the fast MRI and standard MRI patient images did show a decreased signal-to-noise and some blurring in the fast MRI images, as can be seen in the MRI images in Fig. 1. However, these issues did not significantly affect the generated sCT for this cohort of patients.

The clinical study results compare favourably with our previously reported volunteer study [32]. In that, the fast sequence achieved an average body MAE of 33.66 ± 22.04 HU and an average bone MAE of 67.34 ± 34.84 HU. The body DSC was 0.980 ± 0.011 and the bone DSC was 0.916 ± 0.065. In terms of the results for these same anatomical regions, the current patient study resulted in an average MAE for the body of 14.98 ± 2.35 HU, and for the bone of 40.77 ± 5.51 HU, in addition to a body DSC result of 0.985 ± 0.004, and the bone contour producing a DSC result of 0.950 ± 0.018. These HU and anatomical results showed better agreement in the clinical patient study than those achieved in the volunteer study. This may be due to the fast sequence being captured immediately after the standard sequence, as opposed to larger time differences within the volunteer study. From the volunteer study, the isocentre average point dose difference was − 0.14% ± 0.29%, with an average 1%/1mm Gamma pass rate of 99.97 ± 0.03, and an average PTV DVH difference of -0.22% ± 0.21%. From the current patient study, the isocentre average point dose difference was − 0.28% ± 0.16%, with an average 1%/1mm Gamma pass rate of 99.66% ± 0.41%, and an average PTV DVH difference of -0.27% ± 0.18%. The current patient study has comparable dosimetric results, with the slight reduction in gamma pass rate potentially due to the increased treatment plan complexity. The volunteer study treatment plans consisted of a single VMAT arc designed to deliver 78 Gy in 39 fractions, i.e., a conventional 2 Gy per fraction plan. The clinical study treatment plans are for hypofractionated treatment regimes, delivering a much higher dose per fraction compared to the plans in the volunteer study.

As discussed previously in Young et al. [32], from the benchmarking of the standard sequence against CT in Dowling et al. [11], for anatomical factors, a MAE of 40.5, along with a DSC score of 0.91 for bones could be considered for comparing generated sCT back to the standard sequence sCT. An isocentre point dose agreement benchmark of 0.3% ± 0.8% along with a 1%/1mm global gamma pass rate of 95% could also be considered for dosimetric factors. In the current patient study, the fast sequence generated sCT met both these criteria for anatomical and dosimetric agreement.

The current study considers a comparison of sCT with a gold standard sCT. A more appropriate comparison would be true CT, but the study utilised patient data from an MRI-only sub-study within a clinical trial, so capturing an additional CT was not possible or within the study guidelines. However, as the S-sCT approach was previously benchmarked against CT, it could be considered that this was an indirect comparison of sCT to CT. As sCT use for treatment planning and MRI-only radiotherapy becomes more common, further adjustments or improvements in sCT may need to be considered without the availability of a corresponding CT scan. In these cases similar comparisons may be appropriate in assessing suitability of a new sCT.

## Conclusion

In this clinical validation study, the fast sequence, which reduced the required imaging time by approximately a factor of 4, produced an sCT with similar clinical dosimetric results compared to the standard sCT, demonstrating its potential for clinical use for treatment planning.

## Data Availability

Due to the nature of this research, participants of this study did not agree for their data to be shared publicly, so supporting data is not available.

## Appendix

**Table:** Standard SPACE Sequence parameters

Parameter	Standard SPACE Sequence Setting
TE	103 ms
Flip Angle	120 deg
Bandwidth	781 Hz/Px
Field of view	420 – 500 mm
Matrix size	256 × 256
NSA	1.4
Slice thickness	1.6 – 2.0 mm
PAT mode	GRAPPA
